# A Case of Aneurism of the Femoral Artery Cured by Mechanical Compression

**Published:** 1860-05

**Authors:** W. A. Clapp

**Affiliations:** New Albany, Indiana


ART. III.—*A Case of Aneurism of the Femoral Artery cured by Mechanical Compression.* By W. A. CLAPP, M.D., of New Albany, Indiana.

ON the 1st of July, 1858, I was consulted by J. M., a steamboat engineer, twenty-eight years of age, on account of an aneurism of the middle third of the femoral artery. Four months previously, while engaged in his regular occupation, and without any unusual muscular action, he felt a slight snap on the inside of the right thigh, and soon afterwards recognized a small pulsating tumor at the middle of the limb. At the time I saw the case, the tumor had attained the size of a large hen-egg, and was marked by all the characteristic symptoms of an aneurism.

Considering the case a suitable one for compression, on the nineteenth of July I applied two clamps, one at the groin, the other over the femoral artery, about four inches above the tumor, having previously bandaged the limb. The pressure was just sufficient to control the pulsation, and was made alternately with the instruments; the femoral clamp, however, being mostly used so as not to interfere with the return of blood by the saphenous vein. Both clamps were removed at night.

On the following morning the tumor was slightly harder, but the pulsation was undiminished. On the twenty-first, a conical lead weight of four and a half pounds was substituted for the upper clamp, which was not well borne, and the tumor was firmer and slightly reduced in size. For the succeeding seven days the pressure proved very irksome, and was kept up only a few hours each day. Occasional doses of opium were administered to relieve pain, and the tumor had not made any progress toward a cure.

On the twenty-ninth, the treatment having been discontinued for twenty-four hours, on account of pain in the tumor, and a sense of aching and uneasiness in the limb, the pressure was again applied; but instead of being partial, as it had been previously, it was made so as to control the circulation entirely, and the patient was kept fully under the influence of morphia. At 10 o'clock in the evening the tumor was hard and pulseless, the limb was not swollen, and its temperature was but slightly diminished. On the following morning the apparatus was removed.

On the thirty-first, the tumor was considerably diminished, and very hard; but pulsation having returned, the pressure was reapplied. He was directed to have one-fourth of a grain of sulphate of morphia every three hours, and during the evening the pulsation had much diminished.

August 2d.—The thigh clamp, having remained on during the previous night, had caused some excoriation, and the pressure had to be so regulated as to admit of sleep. Pulsation in the tumor was scarcely perceptible, and the affected limb being somewhat colder than the sound one, was wrapped in cotton and flannel. Two days subsequently, twenty drops of tincture of digitalis were ordered every eight hours, on account of the pulsation having increased, and it had greatly diminished up to the eighth instant, when it returned, and was quite forcible in consequence of the clamp having been displaced. On the eleventh, the patient complained so much of inability to stand the pressure that it was removed entirely and again resumed on the sixteenth, he having in the mean while had a severe congestive chill, which readily yielded to treatment.

A great improvement was made in the clamp, a solid india-rubber pad was substituted for the wooden one covered with buckskin hitherto used, and a steel spiral spring added so as to resemble in action Carte's compressor.

17th.—Pulsation ceased entirely at 7 o'clock A.M.; some pain was felt in the aneurism and ankle, but the difference in the temperature of the limbs was slight. The patient expressed himself as being much gratified in the new clamp, he being able to change his position and even to sit up, which he was unable to do before.

18th.—Pulsation was barely perceptible during the day, and up to the twenty-first there was no change, when pulsation having increased, the limb was elevated on an inclined plane.

24th, A.M.—Increased pulsation. P.M.—Since nine o'clock this morning the patient was unable to detect any pulsation; I could occasionally detect a slight throb, but at other times it was imperceptible. Tincture of digitalis was again ordered, of which he has not taken any since the eleventh instant.

27th.—No pulsation; discontinued the digitalis, which has apparently had no effect upon the heart's action.

28th.—Pulsation returned at 7 o'clock A.M., continued two hours, and then ceased.

Compression was kept up until the 4th of September, the aneurism remaining hard and pulseless.

October 4th.—Patient has experienced slight pain throughout the limb during the last month, but it has now ceased, and the tumor is gradually diminishing in size.

March 1st, 1859.—The tumor is very small, pulseless, and hard, and the patient has perfect use of the limb.

The pressure required to entirely control the pulsation in the aneurism, by the lead weight at the groin, was from seven to eight pounds; by the thigh clamp it was equal to fifteen or sixteen pounds.

